# Circulating Immune Cell Profile and Changes in Intravenous Immunoglobulin Responsiveness Over the Disease Course in Children With Kawasaki Disease

**DOI:** 10.3389/fped.2021.792870

**Published:** 2022-02-04

**Authors:** In Su Choi, Mi Ji Lee, Seul A. Choi, Kyung Soon Choi, In Seok Jeong, Hwa Jin Cho

**Affiliations:** ^1^Department of Pediatrics, Chonnam National University Children's Hospital and Medical School, Gwangju, South Korea; ^2^Department of Pediatrics, Miz-I Hospital, Mokpo-si, South Korea; ^3^Department of Cardiothoracic Surgery, Chonnam National University Hospital and Medical School, Gwangju, South Korea

**Keywords:** Kawasaki disease, IVIG responsiveness, immune cell, flow-cytometry, natural killer cells

## Abstract

Kawasaki disease (KD) is an acute, self-limited febrile illness of young children. The etiology of KD remains to be poorly understood. There has been limited research on longitudinal examination of peripheral blood leukocytes for immune profiling particularly in relation to treatment response with intravenous immunoglobulin (IVIG). This study profiles immune cells at the time of diagnosis and over the disease course. In addition, we identified the characteristics of the immune cells in IVIG-responsive patients with KD. We enrolled patients diagnosed with KD between May 1, 2017, and January 1, 2020. Blood was taken at least three times from all enrolled patients: at diagnosis (before IVIG infusion) and immediately and 2 weeks after IVIG infusion. We evaluated the laboratory findings and results of flow cytometry analysis of immune cells at all stages, focusing on CD4^+^ T lymphocytes, CD8^+^ T lymphocytes, CD19^+^ B lymphocytes, granulocytes, classical monocytes, and natural killer (NK) cells. Non-febrile healthy controls (NFCs) and other febrile controls (OFCs) were also enrolled. A total of 68 patients were enrolled and divided into two groups according to IVIG resistance status: IVIG-responsive (*n* = 55) and IVIG-resistant (*n* = 13). The total fever duration was significantly longer in the IVIG-resistant group (9.7 ± 5.3 days) than in the IVIG-responsive group (6.7 ± 3.0 days; *P* = 0.02). There was a significant difference in intermediate CD14^+^CD16^+^ monocytes between KD patients and both NFC and OFCs; they were significantly higher and lower in KD patients than NFC and OFCs, respectively (*P* < 0.001). The levels of all three subtypes of NK cells were significantly lower in KD patients than in both NFC and OFCs (*P* < 0.001). Regarding IVIG responsiveness, CD14^+^CD16^+^ intermediate monocyte levels were significantly lower in the IVIG-resistant group (*P* < 0.001). In addition, CD56^−^CD16^+^ NK cell expression was significantly lower in the IVIG-resistant group than in the IVIG-responsive group (*P* = 0.002). In conclusion, our results suggest CD56^−^CD16^+^N NK cells and CD14^+^CD16^+^ intermediate monocytes might play an essential role in immunopathogenesis of KD. Further studies are warranted to explore the role of these subpopulations particularly for the observed association with coronary artery lesions (CAL) and treatment response.

## Introduction

Kawasaki disease (KD) is an acute, self-limited febrile illness. The etiology of KD is still unknown, but it is, so far, the most common cause of acquired heart disease in children in developed countries (1).

The intravenous immunoglobulin (IVIG) is the classic treatment for KD that reduces the risk of coronary artery lesions (CAL) by 5–25% particularly if it is provided within the first 10 days of disease onset (2). However, 10–20% of patients have IVIG resistance, which is defined as a temperature ≥38.0°C at least 36 h after the end of the initial IVIG infusion; these patients have an increased risk of CALs (1). The mechanism of action of IVIG is not fully understood. Intravenous immunoglobulin plays a role in immune homeostasis by suppressing the activation of innate and adaptive immunity and preventing inflammatory mediators from enhancing anti-inflammatory processes (3, 4). Many researchers have demonstrated the involvement of T lymphocytes, macrophages, and monocytes in KD (5–11). However, there is limited research regarding the immune cell profile throughout the various stages of KD, especially in IVIG-resistant KD patients.

This study profiles immune cells at the time of diagnosis and over the disease course. In addition, we identified the characteristics of these immune cells in IVIG-responsive patients with KD.

## Materials and Methods

We enrolled patients diagnosed with KD who were admitted to a tertiary referral hospital between May 1, 2017, and January 1, 2020. This study was approved by our institutional review board (CNUH-2017-257). All enrolled patients provided written informed consent. We excluded patients with KD transferred from other hospitals after the first dose of IVIG.

All hospitalized patients were treated according to American Heart Association guidelines (1). They received 2 g/kg IVIG and 30–50 mg/kg aspirin, which was reduced to 3–5 mg/kg per day 2–3 days after afebrile status was achieved. We defined IVIG resistance as persistent fever (for 36 h after completion of the first IVIG infusion). Intravenous immunoglobulin-resistant patients underwent laboratory tests and echocardiography on the first day of diagnosis, and the same dose of IVIG was then administered via a second infusion. If the fever persisted for 36 h after completion of the second IVIG infusion, intravenous methylprednisolone pulse therapy (30 mg/kg per dose) was administered for 3 consecutive days. If the fever persisted for 3 days after methylprednisolone infusion, infliximab was infused at a dose of 5 mg/kg.

For all enrolled patients, demographic data were collected including age at diagnosis, sex, body weight, physical examination results, name of the infused drug, and dates of treatment. Blood was taken at least three times from all patients: at diagnosis (before IVIG infusion) and immediately and 2 weeks after IVIG infusion. Patients who were IVIG-resistant also underwent blood tests before the second IVIG infusion; we evaluated the complete blood count (CBC), C-reactive protein (CRP), and pro-brain natriuretic peptide (pro-BNP). In addition, to identify coinfections, samples from the patients routinely underwent polymerase chain reaction for the detection of viruses and mycoplasma infection, as well as blood culture.

Flow cytometry analysis of immune cells, including CD4^+^ T lymphocytes, CD8^+^ T lymphocytes, CD19^+^ B lymphocytes, granulocytes, monocytes, and natural killer (NK) cell subtypes, was done at all testing time points (Navios Flow Cytometry; Beckman Coulter, Brea, CA, USA). The panels included three different monocyte populations, that is, CD14^+^CD16^−^ (classical) monocytes, CD14^+^CD16^+^ (intermediate) monocytes, and CD14^−^CD16^+^ (non-classical) monocytes, as well as three different NK population, that is, CD56^bright^CD16^+^, CD56^dim^CD16^+^, and CD56^−^CD16^+^.

The following monoclonal antibodies and reagents were used for flow cytometry analyses: fluorescein isothiocyanate (FITC)-conjugated anti-cluster of differentiation 3 (CD3), allophycocyanin (APC)-conjugated anti-CD4, phycoerythrin (PE)-conjugated anti-CD8, brilliant violet 510 (BV510)-conjugated anti-CD45 (all from Becton Dickinson Biosciences, San Diego, CA, USA), PerCP/Cy5.5-conjugated anti-CD3, BV421-conjugated anti-CD14, PE-conjugated anti-CD16, APC-conjugated anti CD19, BV510-conjugated anti CD45, PE-Cy7-conjugated anti-CD56, and FITC-conjugated anti-CD66b (all from Becton Dickinson Biosciences). BD Pharm Lyse (a lysis solution) is a buffered, concentrated (10×) ammonium chloride-based reagent (Becton Dickinson Biosciences), and was used to lyse the erythrocytes. Cells were washed with phosphate-buffered saline without calcium and magnesium (Lonza, Walkersville, MD, USA), followed by flow cytometry performed using the Navios flow cytometer (Beckman Coulter).

Non-febrile healthy controls (NFCs) and other febrile controls (OFCs) were also included. The NFCs included those who visited the hospital for routine blood examinations (e.g., for hepatitis B antibodies or anemia), had no history of acute fever within the past 2 months, and were not premature (>35 weeks). The OFC group included those who presented to the emergency department or outpatient clinic for evaluation of acute febrile illnesses such as pneumonia, urinary tract infections, meningitis, and otitis media. Similar to the KD group, the NFCs and OFCs underwent CBC and flow cytometry analyses and were enrolled at comparable ages of 3 months to 7 years.

### Statistical Analyses

Continuous variables are expressed as means ± standard deviations. The independent *t*-test (for normally distributed data) and Mann-Whitney test (for non-normally distributed data) were used to compare continuous variables between the groups. A χ^2^-test was used to compare non-continuous variables. One-way analysis of variance (ANOVA) for normally distributed data and the Kruskal-Wallis test for non-normally distributed data were used to compare continuous variables among the three groups (KD, OFCs, and NFCs). For *post-hoc* analyses, the Tukey method was used. In all analyses, *P* < 0.05 was considered statistically significant. Repeated-measures ANOVA was used to compare serial measurements among timepoints and groups. All analyses were performed using MedCalc Statistical Software (version 19.1; MedCalc Software Bvba, Ostend, Belgium).

## Results

A total of 222 patients were diagnosed with KD and admitted during the study period, 68 of whom were enrolled in this study. We divided those 68 patients into two groups according to IVIG resistance status: IVIG-responsive (*n* = 55) and IVIG-resistant (*n* = 13). The KD groups were compared with the NFCs (*n* = 20) and OFCs (*n* = 15).

### Clinical Characteristics of KD Patients According to IVIG Responsiveness

Among the 68 KD patients, 55 were IVIG-responsive and 13 were IVIG-resistant. The total duration of fever was significantly longer in the IVIG-resistant group (9.7 ± 5.3 days) than in the IVIG-responsive group (6.7 ± 3.0 days; *P* = 0.02). Conjunctivitis (91.2%) was the most common manifestation in enrolled KD patients, followed by rash (89.7%), red lips/tongue (88.2%), edema of the extremities (66.2%), and cervical lymphadenopathy (54.4%). However, no statistical differences between the IVIG-responsive and -resistant groups were observed. The overall incidence of coronary artery dilatation was 10.3%, and it was non-significantly higher in the IVIG-resistant group (*n* = 3, 23.1%) compared with the IVIG-responsive group (*n* = 4, 7.3%). An incomplete form of KD was observed in 24 patients (35.3%): 23 in the IVIG-responsive group and 1 in the IVIG-resistant group (*P* = 0.024).

### Laboratory Findings of KD Patients According to IVIG Responsiveness

All 68 patients underwent laboratory tests; the results of group comparisons at diagnosis are shown in Table 1. The albumin level was significantly lower in the IVIG-resistant compared with the IVIG-responsive group (*P* = 0.002). Other laboratory findings were not different among the groups. A total of five patients were coinfected with either a virus or bacteria. In the IVIG-responsive group, two patients were coinfected with influenza virus, and two with *Mycoplasma*, in addition to KD. In the IVIG-resistant group, one patient was coinfected with influenza virus.

**Table 1:** Clinical characteristics and laboratory findings of Kawasaki disease (KD) patients at the time of diagnosis.

	**Total KD patients** **(***N*** = 68)**	**IVIG-responsive KD** **(***n*** = 55)**	**IVIG-resistant KD** **(***n*** = 13)**	***p*** **-values**
**Clinical characteristics**				
Age, years	2.6 ± 2.2	2.7 ± 2.1	2.3 ± 1.9	0.552
Male, *n* (%)	39 (57.4)	32 (41.8)	7 (46.2)	0.777
Body weight, kg	14.9 ± 6.6	14.8 ± 6.1	15.2 ± 9.4	0.869
Duration of fever since onset, days	6.7 ± 3.0	5.9 ± 1.8	9.7 ± 5.3	0.020
Rash, *n* (%)	61 (89.7)	48 (87.3)	13 (100)	0.177
Conjunctivitis, *n* (%)	62 (91.2%)	49 (89.1)	13 (100)	0.215
Red lips and tongue, *n* (%)	60 (88.2)	47 (85.5)	13 (100)	0.146
Cervical lymphadenopathy, *n* (%)	37 (54.4)	30 (54.5)	7 (53.8)	0.964
Edema of the extremities, *n* (%)	45 (66.2)	34 (61.8)	11 (84.6)	0.185
Coronary arterial lesions, *n* (%)	7 (10.3)	4 (7.3)	3 (23.1)	0.094
Incomplete form of KD, *n* (%)	24 (35.3)	23 (41.8)	1 (7.7)	0.024
Coinfection, *n* (%)	5 (7.4)	4 (7.3)	1 (7.7)	0.006
**Laboratory findings**				
Albumin, g/dl	3.61 ± 0.5	3.7 ± 0.6	3.3 ± 0.5	0.002
Aspartate aminotransferase, IU/L	73.8 ± 96.2	75.5 ± 103.2	66.5 ± 58.5	0.677
Alanine aminotransferase, IU/L	100.9 ± 122.5	101.0 ± 130.0	100.6 ± 86.9	0.990
Blood urea nitrogen, mmol/L	8.6 ± 4.8	8.3 ± 3.9	9.8 ± 7.6	0.494
Creatine kinase, g/dl	169.1 ± 420.5	163.1 ± 41.5	192.2 ± 445.1	0.842
Creatinine, mg/dl	0.3 ± 0.4	0.3 ± 0.4	0.3 ± 0.8	0.771
C-reactive protein, mg/dl	8.3 ± 5.7	8.0 ± 5.6	9.5 ± 6.6	0.449
Hemoglobin, g/dl	11.2 ± 1.8	11.3 ± 1.8	11.2 ± 3.3	0.816
Neutrophil-to-lymphocyte ratio	4.9 ± 4.7	4.2 ± 3.6	7.8 ± 7.1	0.107
Lymphocyte count/mm^3^	3,646.0 ± 2,365.9	3,670.9 ± 2,410.8	3,323.8 ± 2,380.9	0.625
Neutrophil count/mm^3^	10,794.9 ± 7,628.6	9,685.8 ± 5,657.4	15,487.1 ± 12,647.8	0.121
Platelet count/μl	357.9 ± 135.5	349.8 ± 129.2	392.0 ± 200.5	0.379
Potassium, mmol/L	4.3 ± 0.6	4.3 ± 0.6	4.4 ± 0.8	0.631
Pro-BNP, pg/ml	1,473.8 ± 2,644.6	1,045.5 ± 1,657.9	3,222.8 ± 4,524.8	0.145
Total bilirubin, mg/dl	0.7 ± 0.9	0.6 ± 0.8	1.2 ± 1.4	0.159
Total protein, g/dl	6.4 ± 1.1	6.4 ± 1.0	6.3 ± 1.9	0.883
Troponin I, ng/ml	0.03 ± 0.8	0.01 ± 0.9	0.09 ± 1.2	0.215
White blood cells/mm^3^	15,024.4 ± 6,275.9	14,670.2 ± 6,581.9	16,523.1 ± 7,098.5	0.220

*IVIG, intravenous immunoglobulin; KD, Kawasaki disease; pro-BNP, pro-brain natriuretic peptide*.

### Profiles of Immune Cells in KD Patients and Controls at the Time of Diagnosis

All 68 patients, NFCs, and OFCs underwent flow cytometry analyses. CD45^+^ leukocytes, CD3^+^ T lymphocytes, CD4^+^ T lymphocytes, and CD8^+^ lymphocytes were significantly lower in KD patients than NFCs; however, there was no statistically significant difference between KD and OFCs. CD14^+^CD16^−^ “classical” monocyte and CD66^+^ granulocyte levels were significantly higher in KD patients than NFCs; however, there was no significant difference between KD patients and OFCs. By contrast, intermediate CD14^+^CD16^+^ monocyte levels showed a significant difference between KD patients and NFCs and OFCs; they were significantly higher in KD patients compared with NFCs and significantly lower in KD patients compared with OFCs (*P* < 0.001). The levels of all three subtypes of NK cells, namely, CD56^bright^CD16^+−^, CD56^dim^CD16^+^, and CD56^−^CD16^+^ NK cells, were significantly lower in KD patients than both NFCs and OFCs (all *P* < 0.001). The results are shown in Table 2.

**Table 2:** Immune cell profiles in Kawasaki disease patients (IVIG-responsive vs. IVIG-resistant) and comparison with controls at the time of diagnosis.

	**Total KD patients** **(***N*** = 68)**	**NFCs** **(***n*** = 20)**	**OFCs** **(***n*** = 15)**	***p*** **-values**	**IVIG-responsive KD** **(***n*** = 55)**	**IVIG-resistant KD** **(***n*** = 13)**	***p*** **-values**
CD45 leukocyte, %	26.0 ± 13.9^*^	49.9 ± 19.8^*^∫	29.8 ± 5.7∫	<0.001	26.7 ± 3.7	22.8 ± 15.9	0.187
CD3^+^ T lymphocytes, %	12.7 ± 8.3^*^	31.9 ± 12.8^*^∫	15.7 ± 10.5∫	<0.001	12.9 ± 8.1	11.8 ± 9.7	0.742
CD4^+^ T lymphocytes, %	7.7 ± 5.4^*^	19.6 ± 9.1^*^∫	8.7 ± 5.9∫	<0.001	7.8 ± 5.4	6.9 ± 5.6	0.675
CD8^+^ T lymphocytes, %	3.8 ± 2.6^*^	9.6 ± 3.8^*^∫	5.3 ± 5.6∫	<0.001	3.8 ± 2.6	3.4 ± 2.7	0.650
CD4/CD8 ratio	2.2 ± 0.8	2.2 ± 0.8	2.3 ± 1.2	0.089	2.2 ± 0.9	2.2 ± 0.5	0.974
CD14^+^CD16^−^ monocytes, %	3.6 ± 1.9^*^	1.72 ± 0.7^*^∫	3.42 ± 1.6∫	<0.001	6.6 ± 4.1	6.3 ± 4.3	0.855
CD14^+^CD16^+^ monocytes, %	0.68 ± 0.6^*^^ǂ^	0.28 ± 0.2^*^∫	1.09 ± 0.7∫^ǂ^	<0.001	3.7 ± 1.8	3.0 ± 2.4	0.369
CD14^−^CD16^+^ monocytes, %	0.3 ± 0.2	0.2 ± 0.1	0.2 ± 0.1	0.501	0.76 ± 0.6	0.3 ± 0.8	<0.001
CD19^+^ B lymphocytes, %	6.5 ± 4.1	7.6 ± 4.0	4.5 ± 2.7	0.059	0.3 ± 0.5	0.15 ± 0.9	0.124
CD56^++^CD16^+−^ NK cells, %	0.07 ± 0.4^*^^ǂ^	0.3 ± 0.6^*^∫	0.2 ± 0.8∫^ǂ^	<0.001	0.07 ± 0.5	0.05 ± 0.9	0.153
CD56^+^CD16^+^ NK cells, %	1.58 ± 1.4^*^^ǂ^	3.28 ± 1.8^*^	3.55 ± 3.2^ǂ^	<0.001	1.63 ± 1.4	1.35 ± 1.6	0.610
CD56^−^CD16^+^ NK cells, %	0.18 ± 0.4^*^^ǂ^	0.56 ± 2.0^*^	0.46 ± 0.7^ǂ^	<0.001	0.2 ± 0.5	0.08 ± 0.9	0.002
CD66^+^ granulocytes, %	68.5 ± 21.1^*^	34.1 ± 18.2^*^∫	61.4 ± 26.8∫	<0.001	68.5 ± 19.6	68.7 ± 35.9	0.984

*IVIG, intravenous immunoglobulin; KD, Kawasaki disease; NFCs, non-febrile healthy controls; OFCs, other febrile controls. The independent t-test (for normally distributed data) and Mann-Whitney test (for non-normally distributed data) were used to compare continuous variables between groups. One-way analysis of variance (ANOVA; for normally distributed data) and the Kruskal-Wallis test (for non-normally distributed data) were used to compare continuous variables among the three groups (KD, OFCs, and NFCs). For post-hoc analyses, the Tukey method was used. In all analyses, P < 0.05 was considered statistically significant*.

* *KD vs. NFCs, P < 0.05; ∫NFCs vs. OFCs, P < 0.05*;

ǂ *KD vs. OFCs, P < 0.05*.

### Profiles of Immune Cells at the Time of Diagnosis in Patients With KD According to IVIG Responsiveness

CD14^+^CD16^+^ intermediate monocyte and CD14^−^CD16^+^ non-classical monocyte levels were significantly lower in the IVIG-resistant group (*P* < 0.001 and *P* = 0.032, respectively). In addition, CD56^−^CD16^+^ NK cell expression was significantly lower in the IVIG-resistant compared with IVIG-responsive group (*P* = 0.002; Table 2).

### Serial Changes in Immune Cells in KD According to IVIG Responsiveness

**Figure 1:**
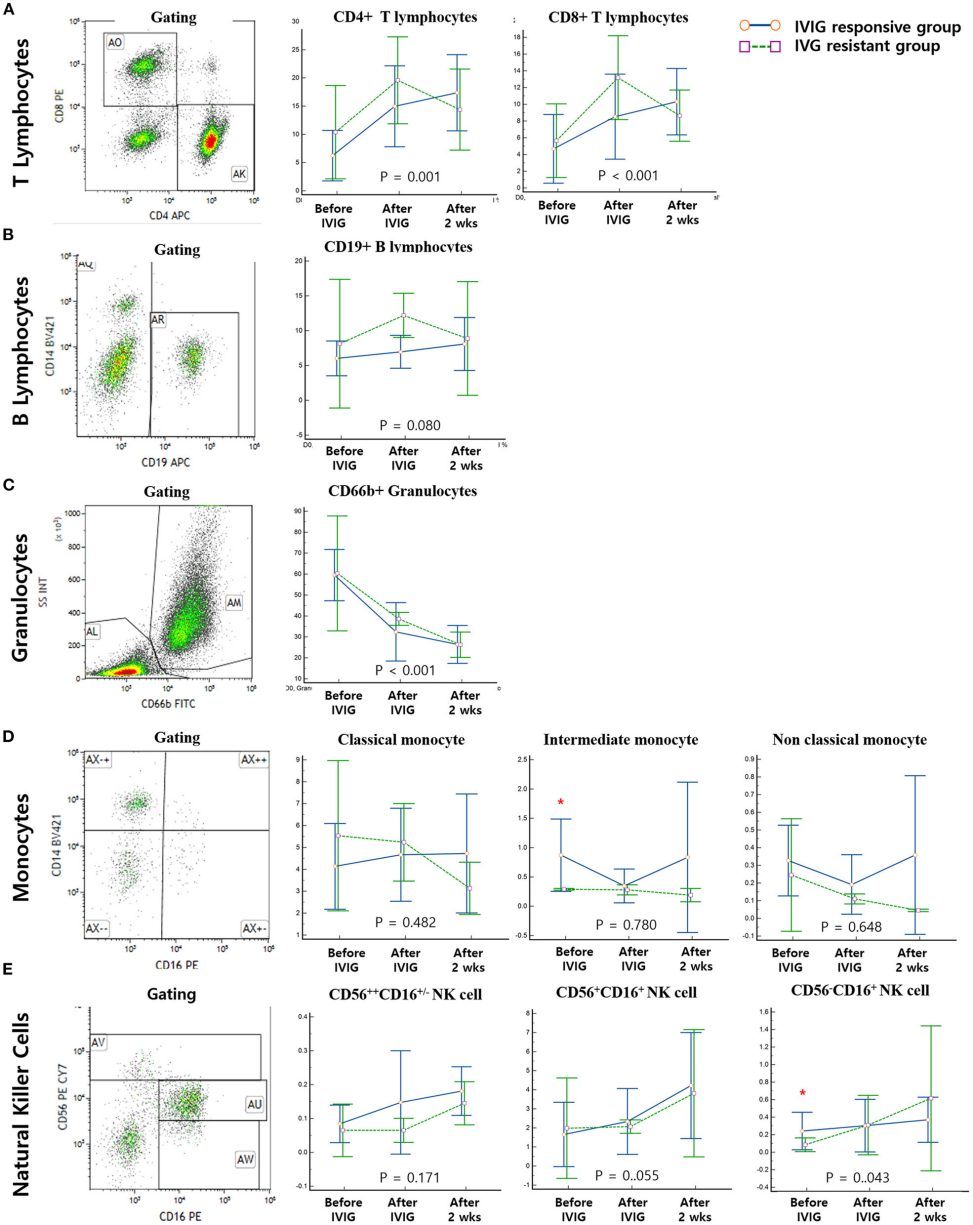
Gating and serial measurements of immune cells. Solid blue lines represent the immunoglobulin (IVIG)-responsive group and dotted green lines represent the IVIG-resistant group. All of the enrolled patients underwent serial measurements of immune cells before IVIG infusion and immediately and 2 weeks after IVIG infusion. **(A)** Both CD4^+^ and CD8^+^ T lymphocytes significantly changed throughout the course of the disease (*P* = 0.001 and *P* < 0.001, respectively); however, no significant differences were seen among groups. **(B)** B-lymphocyte levels did not change significantly and showed no differences among groups. **(C)** Granulocyte expression was significantly decreased over the course of disease (*P* < 0.001), but there was no significant difference among groups. **(D)** No monocyte subtypes changed significantly over the course of the disease; however, there was a significant difference among groups at the timepoint before IVIG infusion. **(E)** Although all subtypes of NK cells showed an increasing trend over the disease course, we found a statistically significant group difference only in CD56^−^CD16^+^ NK cells before IVIG infusion. *Significantly different between IVIG responsive vs IVIG resistant groups on the day of diagnosis (*P* < 0.05).

All patients underwent flow cytometry before IVIG infusion and immediately and 2 weeks after IVIG infusion. Both CD4^+^ and CD8^+^ T lymphocyte expression levels were significantly altered during the disease course (*P* = 0.001 and *P* < 0.001, respectively); however, no significant differences were seen among groups. B lymphocytes did not change significantly and were not significantly different among the groups. Granulocytes were significantly decreased throughout the disease course (*P* < 0.001), but there were no significant differences among the groups. No monocyte subtypes changed significantly throughout the disease course; however, there was a significant difference among groups at the timepoint before IVIG infusion. Although all subtypes of NK cells showed an increasing trend over the disease course, there was a significant group difference only for CD56^−^CD16^+^ NK cells at the timepoint before IVIG infusion (Figure 1).

### Correlation of CD56^−^CD16^+^ NK Cell Levels With Other Clinical and Laboratory Findings

Univariate and multivariate analyses were performed to evaluate the correlation of CD56^−^CD16^+^ NK cell expression with other clinical and laboratory findings. In univariate analyses, fever duration, albumin, alanine aminotransferase (ALT), creatinine, CRP, potassium, total bilirubin, pro-BNP, troponin I, the neutrophil-to-lymphocyte ratio, lymphocyte count, and neutrophil count were significantly correlated with CD56^−^CD16^+^ NK cell expression. All variables with a *p* < 0.1 in univariate analyses were included in the multivariate analyses, in which fever duration, ALT, CRP, lymphocyte count, neutrophil count, and pro-BNP were all significantly correlated with CD56^−^CD16^+^ NK cell expression (Table 3). The correlation between CD56^−^CD16^+^ NK cell expression and clinical manifestations was also evaluated (Table 4). Intravenous immunoglobulin responsiveness, fever duration, CALs, and cervical lymphadenopathy were significantly correlated with the level of CD56^−^CD16^+^ NK cells.

**Table 3:** Results of univariate and multivariate regression analyses of CD56^−^CD16^+^ NK cells: correlations with laboratory findings in IVIG-resistant patients.

	**Univariate analysis**			**Multivariate analysis**		
	**Coefficient**	**SE**	***p***	**Coefficient**	**SE**	***p***
Albumin	0.41	0.17	0.016	0.21	0.17	0.345
AST	−42.5	50.8	0.406			
ALT	−44.9	10.9	0.0001	−0.003	0.0003	0.012
BUN	−0.94	2.49	0.707			
Creatine kinase	−34.3	40.9	0.404			
Creatinine	−0.03	0.02	0.017	2.47	0.64	0.061
C-reactive protein	−5.83	0.99	<0.0001	−0.13	0.01	0.012
Hemoglobin	0.07	0.3	0.820			
NLR	−3.61	0.77	<0.0001	−0.12	0.03	0.066
Lymphocyte count	3,821.59	957.29	0.0002	−0.0001	0.00002	0.022
Neutrophil count	−6,687.1	1,655.9	0.0002	0.00006	0.00001	0.031
Platelets	17.0	91.7	0.853			
Potassium	0.61	0.29	0.044	−0.17	0.10	0.251
Pro-BNP	−1,034.5	355.0	0.0052	0.0001	0.00002	0.031
Total bilirubin	−0.29	0.20	0.153			
Total protein	0.18	0.35	0.614			
Troponin I	−0.03	0.01	0.098	8.08	1.91	0.051
White blood cells	−2,344.3	2,117.4	0.272			

*AST, aspartate aminotransferase; ALT, alanine aminotransferase; BUN, blood urea nitrogen; NLR, neutrophil-to-lymphocyte ratio; Pro-BNP, pro-brain natriuretic peptide*.

**Table 4:** Results of regression analysis of CD56^−^CD16^+^ natural killer cells: correlations with the clinical manifestations of intravenous immunoglobulin unresponsiveness.

	**Coefficient**	**SE**	***p*** **-values**
IVIG responsiveness	−2.98	1.11	0.007
Fever duration, days	−1.85	0.57	0.002
Coronary arterial lesions	−3.31	0.71	<0.001
Cervical lymphadenopathy	−3.33	1.01	0.001
Conjunctivitis	−20.9	8,800.8	0.998
Edema of extremity	−21.9	7,755.9	0.997
Red lip and tongue	−20.9	7,621.7	0.997
Rash	−20.9	8,800.8	0.998

*IVIG, intravenous immunoglobulin*.

## Discussion

This study described the clinical characteristics and distribution of immune cells in patients with KD. Kawasaki disease is a systemic inflammatory disease that affects medium-sized arteries and multiple tissues during the acute febrile phase. Unfortunately, the diagnosis of KD still relies on physical examinations and the exclusion of other etiologies similar to KD based on the principal clinical findings. In this study, we evaluated immune cells circulating in the peripheral blood and also elucidated the differences between IVIG-responsive and -resistant patients. We focused on monocytes and NK cells because flow cytometry analyses showed a significant difference in their levels on the day of diagnosis (before IVIG infusion) between the IVIG-responsive and IVIG-resistant groups. The results on T lymphocytes, B lymphocytes, and granulocytes were similar regardless of the treatment response among patients with KD.

Although many researchers have evaluated the roles of T lymphocytes and monocytes in KD (5–11), how these immune cells differ between non-febrile healthy children and children with febrile infectious is not well-understood. In this study, immune cell profiles were compared between KD patients and NFCs and OFCs, including patients with pneumonia, otitis media, and cellulitis. Whereas, CD4^+^, CD8^+^ T lymphocytes, and classical monocytes showed significant differences between KD patients and NFCs, they showed no differences between KD and OFCs. By contrast, all three subtypes of NK cells and intermediate monocytes showed a significant difference between KD and NFCs and OFCs. Burns et al. (12) recently characterized the various circulating immune cells in acute KD, including myeloid dendritic cells and regulatory T cells, which are associated with diverse antigens that may participate in the pathogenesis of KD.

We further divided KD patients into IVIG-responsive and -resistant groups, because IVIG unresponsiveness can lead to longer-duration fever, resulting in permanent CALs in KD patients. Similar to our previous observations, the intermediate monocyte levels were significantly lower in the IVIG-resistant than IVIG-responsive KD group at diagnosis (before IVIG infusion), in accordance with a previous study (13), but markedly increased thereafter (during the acute phase of KD) (13).

In this study, in addition to intermediate monocytes, we found that CD56^−^CD16^+^ NK cell levels were significantly lower in patients with IVIG-resistant KD at diagnosis (before IVIG infusion). The role of NK cells in the pathophysiology of KD is not well-understood. In a recent report, NK levels were similar across KD patients (12); however, the study did not include the subtype of NK cells. Interestingly, CD56^bright^ NK cells were reported to accumulate within human atherosclerotic lesions, and the authors suggested a possible contribution of NK cells to plaque instability (14). However, involvement of these cells in inflammatory vasculitis is not known, and to our knowledge, the results presented on NK cells are novel.

CD56^−^CD16^+^ NK cells are naturally a small population of peripheral blood lymphocytes. The functional characteristics of these cells have been studied in patients with viral diseases, such as human immunodeficiency virus (15–17). The role of NK cells in the course of KD remains to be fully elucidated, especially in the context of IVIG treatment. In this study, although CD56^−^CD16^+^ NK cell levels were significantly different between the IVIG-responsive and -resistant groups at the time of diagnosis (before IVIG infusion), they correlated with IVIG responsiveness, fever duration, CALs, and cervical lymphadenopathy. CD56^−^CD16^+^ NK cell expression was also related to pro-BNP, CRP, and ALT levels and the lymphocyte and neutrophil counts. Of note, the relatively low level of CD56^−^CD16^+^ NK cells before IVIG infusion in IVIG-resistant KD patients increased to match the level of CD56^−^CD16^+^ NK cells in IVIG-responsive KD patients after IVIG infusion; a similar pattern was seen 14 days after IVIG infusion in IVIG-responsive KD patients. While IVIG is usually the first-line treatment for KD, non-responsiveness to IVIG is a challenge in clinical practice. Many tools have been developed to predict IVIG unresponsiveness and minimize the complication of CALs (18–22). However, these instruments are not ideal for every patient (23–26). This highlights the need for a deeper understanding of the pathophysiology of KD and for biomarkers that can be used to identify children at increased risk of coronary artery abnormalities. This could aid the selection of patients appropriate for anti-inflammatory treatment and IVIG. Regarding NK receptor expression and function, we presume that the relatively low expression CD56^−^CD16^+^ NK cells in the IVIG-resistant group might play a role in the pathophysiology of KD and response to IVIG treatment. Additional studies are needed to verify the KD specificity of CD56^−^CD16^+^ NK cells, especially in the context of IVIG treatment, and to elucidate the molecular mechanisms underlying the dysregulation of NK receptor expression and function.

This study had some limitations. First, the size of the KD patient population was relatively small, especially with respect to those who were IVIG-resistant. A large number of patients were excluded because they were transferred from other hospitals after the first dose of IVIG. As we did not see a change in patients who had already received IVIG infusion before arrival, we had to exclude them. Second, only the number/proportion of immune cells was assessed; no functional studies were performed. Third, as the age range was diverse, we did not adjust the lymphocyte data to reference values; instead, we used percentage rather than absolute values. Also, we compared the lymphocyte subgroups with two types of controls, OFCs and NFCs, of comparable age. In addition, we focused on changes in lymphocytes and other immune cells in the acute/subacute and convalescent stage rather than absolute cutoff values. This limited the clinical utility of our study, as we did not provide cutoff values.

In conclusion, CD14^+^CD16^+^ intermediate monocytes, as well as CD56^−^CD16^+^ NK cells, might play an essential role in disease outcomes and response to treatment with IVIG in KD patients. Further studies based on large sample sizes are warranted to elucidate the mechanisms involved and determine if these cell populations can be of value as predictive markers for treatment response.

## Data Availability Statement

The raw data supporting the conclusions of this article will be made available by the authors, without undue reservation.

## Ethics Statement

The studies involving human participants were reviewed and approved by Chonnam National University Hospital, CNUH-2017-257. Written informed consent to participate in this study was provided by the participants' legal guardian/next of kin.

## Author Contributions

HC and IJ conceptualized and designed the study, drafted, reviewed, and revised the manuscript. IC and ML reviewed and revised the manuscript. SC and KC contributed to the data analyses. All authors approved the final manuscript as submitted and agree to be accountable for all aspects of the work.

## Funding

This work was supported by the BCR120006, BCRI19045, and BCRI19255 from the Biomedical Institute of Chonnam National University Hospital and also by the NRF-2019R1D1A3A03103899 and NRF-2020R1F1A1073921 from the Korean National Research Foundation.

## Conflict of Interest

The authors declare that the research was conducted in the absence of any commercial or financial relationships that could be construed as a potential conflict of interest.

## Publisher's Note

All claims expressed in this article are solely those of the authors and do not necessarily represent those of their affiliated organizations, or those of the publisher, the editors and the reviewers. Any product that may be evaluated in this article, or claim that may be made by its manufacturer, is not guaranteed or endorsed by the publisher.
