# Supplementary material for: Circulating Immune Cell Profile and Changes in Intravenous Immunoglobulin Responsiveness Over the Disease Course in Children With Kawasaki Disease
Source: Front Pediatr. 2022 Feb 4;9:792870. doi: 10.3389/fped.2021.792870 (PMC8855096; doi:10.3389/fped.2021.792870)
Supplement: Supplementary file 1 [file Table_1.DOCX]

|  | **Human antibodies** | **Company** |
| --- | --- | --- |
| **T lymphocytes** | **FITC Mouse Anti-Human CD3** | **BD Bioscience** |
|  | **APC Mouse Anti Human CD4** | **BD Bioscience** |
|  | **PE Mouse Anti-Hyman CD8** | **BD Bioscience** |
|  | **BV510 Mouse Anti-Human CD45** | **BD Bioscience** |
| **Monocytes, granulocyte, B lyomphocyte, NK Cell subtypes** | **BV421 Mouse Anti-Human CD14** | **BD Bioscience** |
|  | **PerCP/Cy5.5 Mouse anti-human CD3** | **BD Bioscience** |
|  | **BV510 Mouse Anti-Human CD45** | **BD Bioscience** |
|  | **PE-Cy7 Mouse Anti-Human CD56** | **BD Bioscience** |
|  | **PE Mouse Anti-Human CD16** | **BD Bioscience** |
|  | **APC Mouse Anti-Human CD19** | **BD Bioscience** |
|  | **FITC Mouse Anti-Human CD66b** | **BD Bioscience** |

Supplementary Table 1: Antibodies for flow cytometric analyses
